# Analysis of In Situ Electroporation Utilizing Induced Electric Field at a Wireless Janus Microelectrode

**DOI:** 10.3390/mi15070819

**Published:** 2024-06-25

**Authors:** Haizhen Sun, Linkai Yu, Yifan Chen, Hao Yang, Lining Sun

**Affiliations:** 1 School of Mechanical and Electric Engineering, Soochow University, Suzhou 215299, China; 20235229001@stu.suda.edu.cn (L.Y.); yfchen1@stu.suda.edu.cn (Y.C.); lnsun@suda.edu.cn (L.S.); 2 Jiangsu Provincial Key Laboratory of Advanced Robotics, School of Mechanical and Electric Engineering, Soochow University, Suzhou 215123, China

**Keywords:** in situ electroporation, Janus particle-based microelectrode, localized electric field, cell membrane permeabilization

## Abstract

In situ electroporation, a non-invasive technique for enhancing the permeability of cell membranes, has emerged as a powerful tool for intracellular delivery and manipulation. This method allows for the precise introduction of therapeutic agents, such as nucleic acids, drugs, and proteins, directly into target cells within their native tissue environment. Herein, we introduce an innovative electroporation strategy that employs a Janus particle (JP)-based microelectrode to generate a localized and controllable electric field within a microfluidic chip. The microfluidic device is engineered with an indium tin oxide (ITO)-sandwiched microchannel, where the electric field is applied, and suspended JP microelectrodes that induce a stronger localized electric field. The corresponding simulation model is developed to better understand the dynamic electroporation process. Numerical simulations for both single-cell and chain-assembled cell electroporation have been successfully conducted. The effects of various parameters, including pulse voltage, duration medium conductivity, and radius of Janus microelectrode, on cell membrane permeabilization are systematically investigated. Our findings indicate that the enhanced electric intensity near the poles of the JP microelectrode significantly contributes to the electroporation process. In addition, the distribution for both transmembrane voltage and the resultant nanopores can be altered by conveniently adjusting the relative position of the JP microelectrode, demonstrating a selective and in situ electroporation technique for spatial control over the delivery area. Moreover, the obtained differences in the distribution of electroporation between chain cells can offer insightful directives for the electroporation of tissues or cell populations, enabling the precise and targeted modulation of specific cell populations. As a proof of concept, this work can provide a robust alternative technique for the study of complex and personalized cellular processes.

## 1. Introduction

The intracellular delivery of molecules, such as DNA probes and plasmids, has developed into a robust fashion for numerous biomedical issues [1,2,3]. The essence of efficacious intracellular delivery lies in the capability of molecules to pass through the cell membrane and enter the inside of cells. A variety of techniques, such as viral transfection [4,5], chemical modifications [6,7], mechanical contact [8,9,10], electrical excitation [11,12], and thermal membrane disruption [13,14,15], have been developed to perform cargo delivery into the intracellular environment. Among these, electroporation, a highly efficient non-invasive transfection technique, has emerged as a robust tool able to deliver various types of molecules into cells, offering higher transfection efficiency with lower cytotoxicity for studying gene functions, protein expression, and drug screening [16,17]. 

Electroporation is a bioelectrochemical process where a short, intense electric field is applied to cells, leading to the formation of transient aqueous pores in the lipid bilayer, thus enabling the transmembrane transport of molecules. Conventional bulk electroporation (BEP) is a transfection technique that employs high-voltage electrical pulses to induce reversible permeabilization of the membrane across a large population of cells, facilitating the uptake of extracellular molecules [18,19]. However, the applied high voltage may induce pH changes and Joule heating in the cell culture environment, which would reduce cell viability. In addition, BEP exhibits limitations in achieving transfection uniformity and lacks the capability for in situ single-cell electroporation. To circumvent these limitations, a plethora of microscale electroporation methodologies has been burgeoning in the field, such as flow-through electroporation, nanostructure-based electroporation, and microelectrode array-based electroporation. These techniques are executed within microenvironments and are facilitated by microelectrodes integrated onto microfluidic platforms, thereby significantly augmenting the precision and efficiency of transfection processes [17,20]. Flow-through electroporation typically necessitates the utilization of flow focusing or narrow channels to form a cell stream that traverses the electrode-pair region where the electric pulse is applied, leading to the occurrence of electroporation [21,22,23]. Despite its high throughput, this manipulation cannot be precisely controlled to maintain high cell viability due to the prolonged duration of electric field exposure experienced by the cells. Furthermore, this approach is also constrained in terms of localized electroporation and the variability in pore location. Nanostructure-based electroporation consistently relies on patterned nanochannels [24,25,26,27] or nanostraws [28,29], where cells make direct contact and a localized electric field emerges, which has been demonstrated to be efficacious for in situ cell electroporation and practical cellular applications [30]. However, its reliance on complex device fabrication escalates the cost, and the pore position is confined to the contact region, thereby imposing limitations on its versatility and applicability. The microelectrode array-based electroporation is a sophisticated technique that initially leverages electrodynamic forces to facilitate single-cell trapping [31,32,33,34]. Subsequently, the electric signal is transformed into a pulse signal, which induces the disruption of the cell membrane and enables the uptake of external molecules. Beyond necessitating intricate electrode network fabrication, the process also demands a stringent microenvironment for the execution of cell trapping, thereby increasing the operational complexity. The aforementioned electroporation methodologies are predicated on the conventional paradigm of utilizing driving electrodes, wherein the direct application of an electric field across electrode plates induces the electroporation phenomenon in cells. Various numerical models have been developed to evaluate the electroporation performance. Specifically, a double-layered spherical cell model considering conductivity change in the electroporation process was built, which can reflect the more accurate spatiotemporal evolution of the electroporation process [35,36]. On this basis, a single-cell electroporation model considering pore area ratio and area weight was developed to investigate the dynamics of electroporation [37]. A multiphysics approach combining the electric field, ion transport, membrane deformations, and membrane permeabilization was established to predict the electrotransfection performance [38]. However, the exploration of cutting-edge electroporation techniques that incorporate novel numerical modeling is currently underrepresented in the scholarly discourse. Therefore, the present work aims to establish a novel electroporation model incorporating the existing model and induced electric field to guide the design of a localized electroporation platform.

In this work, we exploit a unique wireless electrode, namely JP microelectrode, to achieve in situ cell electroporation. The localization mechanism of JP for capturing cells can be achieved through the special design of JP. JPs are microparticles with two different materials or functionalities, typically in a spherical structure, with one half exhibiting one property and the other half exhibiting another. When capturing cells, their movement in solution can be controlled using external forces or flows to bring them close to the cells. For example, external magnetic fields can be applied to manipulate the motion of magnetic JPs and change their relative positions with cells. Additionally, microscopy can be employed to monitor and observe the interaction between JPs and cells in real time, further aiding in the control of the capture process. We have engineered an indium tin oxide (ITO)-based microfluidic chip integrated with suspended JP microelectrodes. This platform enables the precise positioning of the JP microelectrode in proximity to target cells, followed by its activation to induce a voltage through an external pulse signal, thereby executing electroporation [39,40]. A novel numerical model is developed to better understand the dynamic electroporation process utilizing the induced electric effect. Our findings indicate that the local electric field is significantly enhanced in the vicinity of the JP microelectrode, which plays a pivotal role in the electroporation phenomenon. The pore formation can be precisely controlled by adjusting the spatial relationship between the JP microelectrode and the cells, thereby enabling a selective and localized electroporation technique for spatial regulation of the delivery region. Additionally, we have successfully characterized the electroporation profiles of paired cells, delineating the disparities in pore distribution between them. In combination, the JP microelectrode-based electroporation technique is capable of expanding the toolkit for cell transfection and holds great potential in broadening cellular assays.

## 2. Model Building and Simulation Design

### 2.1. Numerical Modeling

#### 2.1.1. Establishment of Cell Electroporation Model 

**Figure 1:**
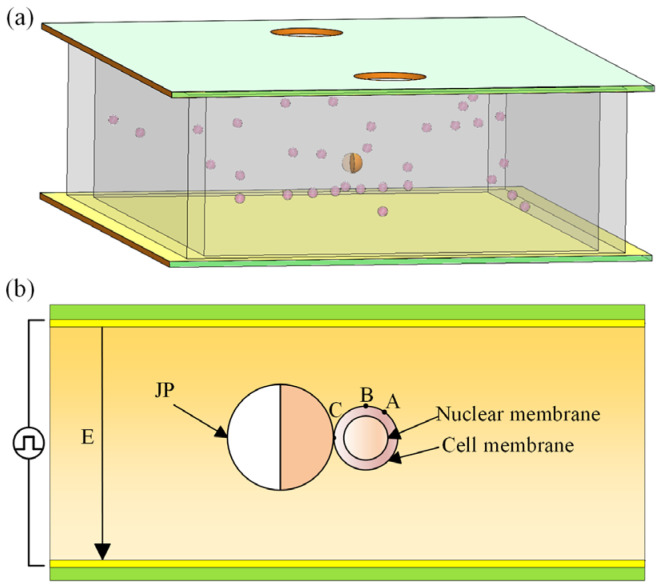
A schematic diagram of electroporation simulation. (**a**) 3D geometrical model, the smaller the volume of cells, the larger the volume of JP. (**b**) Two-dimensional cross-section of the simulation setup, a schematic illustration of electroporation after the JP captures the cell.

In this work, the electroporation dielectric model is established by using COMSOL Multiphysics software 6.0. Figure 1a is a schematic of the 3D simulation model, where the smaller are the cells and the larger is the JP. A large number of dispersed cells are suspended in extracellular fluid. Figure 1b shows the two-dimensional cross-section of the simulation model. In order to consider the spherical single-cell multilayer dielectric model of the cell, the current module is used for transient analysis. The electric field intensity in the simulation is applied by the upper and lower electrode plates. In the figure, the electrode plate, extracellular suspension, Janus particle (JP), and cell are shown from outside to inside. The cell model is a spherical single-cell multilayer dielectric model, which consists of the cell membrane, cytoplasm, and nuclear membrane from outside to inside. The length of the electrode plate is 500 µm, and the distance between the upper and lower plates is 50 µm. One electrode is grounded and the other electrode is connected with a pulse signal. The boundaries of all areas where the cells are located are electrically insulated to ensure that the simulated electric field is a uniform one. 

Between the two plates, a pulse signal of a specific amplitude and a specific width is applied. The JP captures the cells in the extracellular suspension, changes the surrounding electric field itself to form an induced electric potential, forms an electric field of a certain intensity around itself, and generates a voltage on the cell surface. When the electric field intensity reaches a certain level, the cell will electroporate. Three markers A, B, and C are selected on the cell surface for subsequent electroporation effect analysis. In this work, the grid of the simulation model is triangular, and the coefficient boundary partial differential equation in the PDE module is used to establish a dynamic numerical model of electroporation, which analyzes the electroporation effect of the inner and outer membrane, including the transmembrane voltage, pore density, and the perforation region.

The idea of the above numerical model of electroporation using a partial differential equation is as follows: When the transmembrane potential corresponding to the cell membrane and nuclear membrane reaches the perforation threshold, the pore density of the inner and outer membranes will increase, resulting in an increase in the conductivity and permeability of the cell membrane. At this time, more current flows through the formed micropores, thus increasing the transmembrane current density *J*(*t*). *J_EP_*(*t*) can be used to characterize the increment of *J*(*t*) [37,41].

(1) $$J(t)=\frac{\sigma_{mem0}}{d_{mem}}(\psi_{i}-\psi_{o})+\frac{\varepsilon_{0}\varepsilon_{m}}{d_{mem}}\frac{\partial (\psi_{i}-\psi_{o})}{\partial t}+J_{EP}(t)$$

where *σ_mem_*_0_ is the conductivity of the membrane before perforation; *ε*_0_ is the dielectric constant of vacuum; *ε_m_* is the relative dielectric constant of the cell membrane. *d_mem_* is the thickness of the cell membrane. *ψ_i_* is the membrane medial potential. *ψ_o_* is the outer membrane potential.

(2) $$J_{EP}(t)=i_{EP}(t)N(t)$$

(3) $$i_{EP}(t)=(\psi_{i}-\psi_{o})\sigma_{p}\pi r_{p}^{2}\frac{A}{h}$$

where *i_EP_*(*t*) is the current flowing through a single hole; *N*(*t*) is the perforation density of the film. *σ_p_* is the void conductivity; *r_p_* is the radius of the hole *N*(*t*), which can be solved by Equation (4).

(4) $$\frac{dN(t)}{dt}=\alpha e^{{\left(\frac{\Delta \psi (t)}{U_{EP}}\right)}^{2}}(1-\frac{N(t)}{N_{0}}e^{-q(\frac{\Delta \psi (t)}{U_{EP}}{)}^{2}})$$

By substituting Equations (3) and (4) into Equation (2):

(5) $$J_{EP}(t)=\frac{\sigma_{men0}(t)(\psi_{i}-\psi_{o})}{d_{men}}+\frac{\varepsilon_{0}\varepsilon_{m}\partial (\psi_{i}-\psi_{o})}{\partial t}$$

where *σ_mem_*(*t*) is the conductivity of the cell membrane after perforation.

#### 2.1.2. Induced Potential of JP Microelectrode

The electric potential on the JP can be achieved by solving the Laplace equation [42]:

(6) $$\nabla \cdot (\sigma E)=-\sigma \nabla^{2}\phi =0$$

assuming the electric conductor has a constant conductivity *σ*.

The JP can be assumed to act as a capacitor in series with a diffuse-layer capacitor; the total induced double-layer (IDL) capacitance is C_0_ = C_S_C_D_/(C_S_ + C_D_) = C_D_/(1 + *δ*). The voltage across the diffuse layer capacitor only occupies a portion of the total double layer voltage *ψ*_D_ = Δ*ϕ*/(1 + *δ*), where C_D_ = *ε*/*λ*_D_ is the capacitance of the diffuse layer, and C_S_ is the capacitance of the Stern layer. The ratio of the diffuse layer capacitance to that of the Stern layer is *δ* = C_D_/C_S_, while *ε* is the permittivity (*ε* = 7.08 × 10^−10^ F/m) and *λ*_D_ = (*Dε*/*σ*)^1/2^ = 37.6 nm is the Debye screening length, where D = 2 × 10^−9^ m^2^/s is the bulk diffusivity.

A capacitance-like boundary condition closes the equivalent RC circuit and the normal current from the bulk charges of the diffuse layer [43,44].

(7) $$C_{0}\frac{d\psi_{0}}{dt}=-\sigma \hat{n}\cdot \nabla \phi =\sigma E_{n}$$

Using complex amplitudes, the above condition can be written as follows:

(8) $$jwC_{0}\frac{\tilde{\phi }-{\tilde{\phi }}_{0}}{1+\delta }=\sigma \hat{n}\cdot \nabla \tilde{\phi }$$

where $\tilde{\phi }$ is the potential in the bulk outside the JP, and ${\tilde{\phi }}_{0}$ is the potential at the metal surface. The boundary condition at the insulating surface can be given by [45]:

(9) $$\frac{\partial \phi }{\partial y}=0$$

Compared with the traditional electroporation simulation, a new electroporation method is adopted: The electroporation phenomenon is induced by using JP. The above geometric model and the numerical model of electroporation are established. Through simulation and comparative analysis, the electroporation effects of JP at different positions and sizes, cells at different positions and sizes, as well as different electrical conductivities, are studied under varying pulse amplitudes and widths. The study aimed to investigate the impact of these variables on the efficiency of electroporation. This research explores the complex interplay between the physical properties of the Janus particles, the cellular characteristics, and the electrical parameters in order to enhance our understanding of the mechanisms underlying electroporation.

## 3. Simulation Results and Discussions

### 3.1. Characterization of Induced Electric Field 

**Figure 2:**
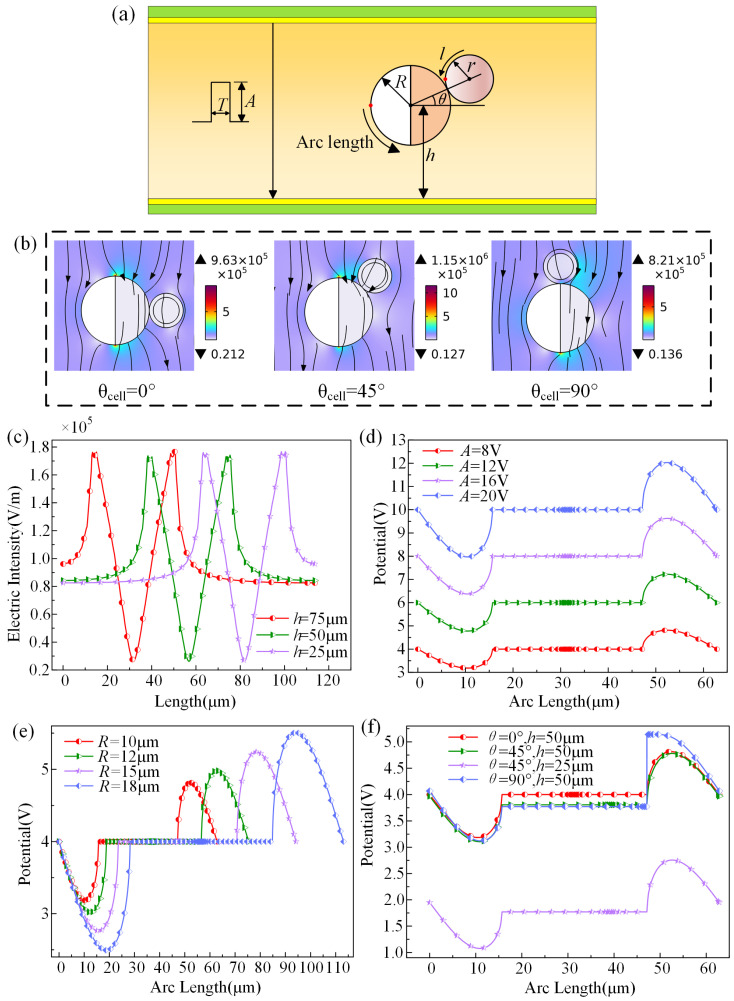
(**a**) Schematic diagram of model parameters. (**b**) Electric field distribution diagram when the cell angles are 0°, 45°, and 90°, respectively. (**c**) Surrounding field strength curves when the JP is located at distances of 25 µm, 50 µm, and 75 µm from the lower electrode plate. (**d**) Induced potential curves of the Janus sphere at pulse voltages of 8 V, 12 V, 16 V, and 20 V, respectively. (**e**) Induced potential curves of the JP with radii of 10 µm, 12 µm, 15 µm, and 18 µm. (**f**) Induced potential curves of the Janus sphere at capture cell positions of 0°, 45°, 90°, and at a distance of 25 µm from the lower electrode plate.

The relevant results obtained through modeling and simulation of JP and cells are shown in Figure 2. Figure 2a depicts a schematic representation of some parameters during the simulation process, serving to illustrate the variables involved in the simulation. Electroporation simulations are conducted on cells captured by the JP at various positions under a pulsed electric field of 10 μs and 8 kV/cm. The deviation angles of cell positions relative to the JP are 0°, 45°, and 90°. Figure 2b illustrates the distribution of electric fields under three different conditions, showing that the inherent conductivity of JP causes a deviation in the parallel electric field between the electrode plates in its vicinity. This results in the convergence of the parallel electric field on the conductive side of the Janus sphere surface above, and the reversal of the symmetrical route of the field convergence lines below, gradually reverting to a parallel electric field near the lower electrode plate. Variations in cell capture locations lead to differences in the deviation of surrounding electric field lines, resulting in varying electric field strengths on the surface of the Janus sphere. As observed in the figure, when the capturing cell position is at 45°, the electric field intensity on the right side of the Janus sphere is the highest, followed by the position at 0°, and the position at 90° exhibiting the lowest intensity.

Considering the substantial mass of the JP itself, the capture position of cells by the JP between parallel electrode plates will experience some changes. As depicted in Figure 2c, the graph illustrates the fluctuation in surrounding electric field intensity at varying distances from the center of the JP to the electrode plate. Here, the arc length denotes as the circular line counterclockwise from the leftmost point for both the JP electrode and cells. At *h* = 50 μm, indicating the JP positioned centrally on the electrode plate, an observation of symmetrical electric field distribution around the JP is apparent. Following the application of pulses to the upper and lower electrode plates, the electric field gradually intensifies towards the JP from both ends, with the peak intensity observed as it nears the upper and lower vertices. Subsequently, a sharp decline in electric field intensity is noted along the JP’s surface to its minimal value at the right vertex of the conducting part. At distances of *h* = 25 μm and *h* = 75 μm, a similar trend in electric field intensity variations is observed, manifesting a near-identical distribution pattern on the JP’s surface as to when *h* = 50 μm. The distinguishing factor lies in the varying distances from the upper and lower electrode plates, leading to differences in the initial field strength. A larger distance from the electrode plate results in lower initial field strength with a slower acceleration rate, while closer proximity generates higher initial field strength and a faster rate of increase.

To delve deeper into the impact of the JP on electroporation efficacy, an investigation is conducted on the self-induced potential of the JP concerning the applied pulse amplitude, JP size, and positional variations, as illustrated in Figure 2d–f. As depicted in Figure 2d, as the JP is positioned at the midpoint of the electrode plate, the induced electric potential on the JP’s surface alters in accordance with the applied voltage amplitude. Given that one-half of the JP acts as a conductor, resulting in a uniform induced potential on its surface at the midpoint of the electrode plate, it can be deduced that this induced potential equates to half of the applied voltage. Conversely, the non-conductive insulator segment of the JP determines its surface-induced potential based on the electric field’s location on the surface. The graph illustrates that the electric potential of the conductive portion remains constant, representing half of the applied pulse amplitude at the electrode plate’s midpoint. As the insulator portion of the JP reaches the upper section, the surface-induced potential increases as it approaches the upper electrode plate, eventually aligning with the conductive isopotential area. Subsequently, the surface-induced potential reverts to half of the pulse amplitude. Furthermore, the applied pulse amplitude influences the rate of induced potential alteration with distance. In Figure 2e, the induced potential curves demonstrate variations with the changes in the size of the JP. When the pulse voltage magnitude remains constant, an increase in the radius of the JP leads to a corresponding increase in the amplitude of induced potential on the insulating side. Although the shapes of the induced potential curves remain consistent across different radii, they amplify with increasing radius. This behavior aligns with the patterns described in Figure 2d. Figure 2f explores the induced potential of cells captured by the JP at various locations. The graph highlights that the induced curve aligns with the aforementioned pattern when the cell capture angle is 0° and 45°. Notably, when the JP nears the lower electrode plate, the constant voltage of its conductive side adjusts based on the center-to-bottom distance, yet remains unaltered for the insulator side. At a capture angle of 90°, an electroporation effect occurs near the cell’s apex, causing a deviation in voltage on the insulator side compared to the conductor apex. Likewise, at 45°, due to the asymmetric cell positioning, the initial voltage magnitude impacting the insulator side’s induced potential differs from that of the conductive side.

### 3.2. Evolution of Transmembrane Voltage during Electroporation

**Figure 3:**
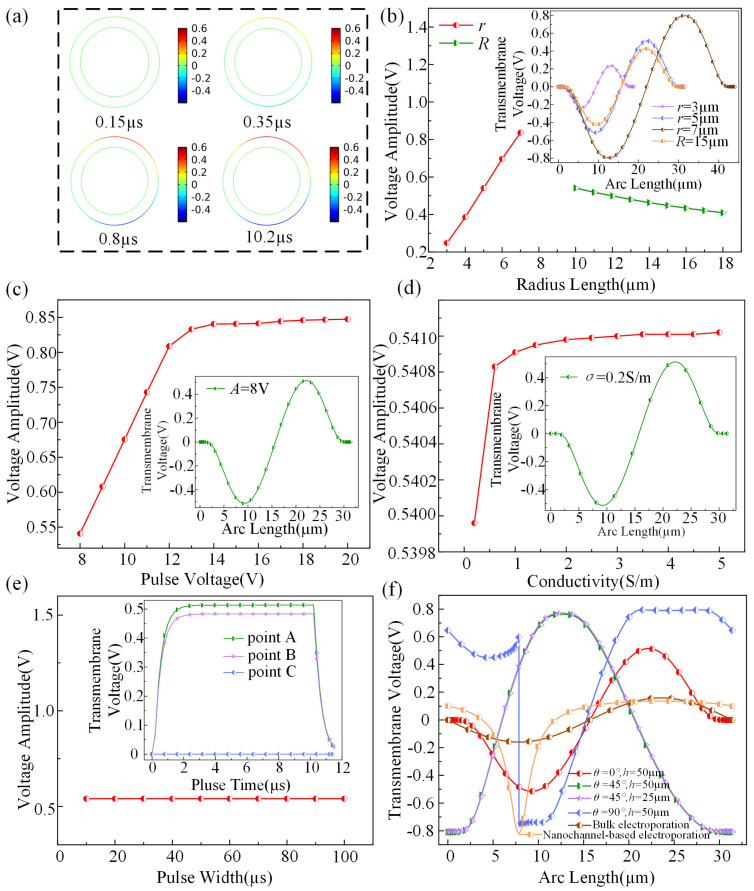
(**a**) Distribution of transmembrane voltages across cells at time points of 0.15 µs, 0.35 µs, 0.8 µs, and 10.2 µs. (**b**) Relationship curve between transmembrane voltage amplitude on the cell surface and the sizes of Janus spheres and cells. (**c**) Relationship curve between transmembrane voltage on the cell surface and pulse voltage amplitude. (**d**) Relationship curve between transmembrane voltage on the cell surface and conductivity. (**e**) Relationship curve between transmembrane voltage on the cell surface and pulse width. (**f**) Transmembrane voltage curves capturing cell positions at 0°, 45°, and 90° angles relative to Janus spheres and at a distance of 25 µm from the electrode plate.

For a more in-depth examination of the electroporation impact on cells induced by JPs, this study categorically analyzed the transmembrane voltage of cells. It investigated the effects of various parameters, including the radius of the JP, cell radius, pulse voltage, conductivity, pulse width, and cell location, on the distribution of transmembrane voltage. These analyses are presented in Figure 3. 

In order to depict the variations in transmembrane potential more vividly, this paper includes time points. Figure 3a shows the temporal distribution of the transmembrane potential of the cell under a pulse voltage of 8 V and pulse width of 10 µs. At 0.15 µs, no pulse voltage is applied, and there is no voltage distribution on the cell surface. At 0.35 µs, the pulse voltage is applied, resulting in a weak voltage generated on the upper and lower surfaces of the cell. By 0.8 µs, the transmembrane potential on the cell surface is still increasing, reaching 0.4 V at the maximum point. At 10.2 µs, the transmembrane potential on the cell surface had stabilized, reaching its maximum value. It can be observed that the voltage surge on the cell surface after pulse voltage application rapidly reaches a maximum before stabilizing till the pulse duration ends. The polarity of voltage distribution on the upper and lower cell surfaces is symmetric, with positive values on the upper half and negative values on the lower half. The correlation between cell surface transmembrane voltage amplitude and JP size, as well as cell size, is depicted in Figure 3b. From the figure, it can be observed that, under constant parameters, the transmembrane voltage of the cell increases with the increase in cell radius within the range of 3 µm to 7 µm. Conversely, when the radius of JP ranges from 10 µm to 18 µm, the amplitude of transmembrane voltage decreases with the increase in JP radius. The curves illustrating the changes in transmembrane voltage of the cell at *r* = 3 µm, *r* = 5 µm, and *r* = 7 µm are shown in the graph. Additionally, when the cell radius is kept constant, comparisons are made with different JP radii of 10 µm and 15 µm, revealing that the amplitude of the transmembrane voltage of the cell decreases with the increase in JP radius, consistent with the previously described pattern.

Figure 3c showcases the correlation between transmembrane voltage and pulse voltage amplitude. Starting with an initial voltage of 8 V, subsequent increments to 20 V display a proportional increase in transmembrane voltage, aligning with the findings of Fei Guo’s research. After reaching a voltage value of 14 V, the amplitude of transmembrane voltage growth slows down and gradually stabilizes. Figure 3d illustrates the correlation between transmembrane voltage and conductivity. The data indicate an increase in conductivity from 0.2 S/m to 5 S/m, yet this variation exerts minimal influence on the amplitude of the final transmembrane voltage. Moving on to Figure 3e, which portrays the relationship between transmembrane voltage and pulse width, an investigation reveals that as the pulse width extends from 10 µs to 100 µs, there is no consequential impact on transmembrane voltage amplitude. Analyzing transmembrane voltage changes over time at three designated points A, B, and C on the cell surface, it is observed that with increasing pulse signals, both points A and B experience a rapid surge in transmembrane voltage from rest potential over a short duration. By 2.5 µs, the voltage peaks and stabilizes, remaining constant throughout the pulse duration before swiftly dropping back to 0 V post-pulse. In contrast, point C, situated at the interface between the JP and the cell, maintains a constant 0 V transmembrane voltage during electroporation. Figure 3f unveils the relationship between transmembrane voltage and the capture cell’s position, taking into account a scenario where the capture angle is set at 0°. As the angle shifts to 45°, a loss of symmetry in transmembrane voltage distribution along the cell surface is observed. The amplitude of positive and negative transmembrane voltage peaks around 0.8 V, significantly higher than under normal conditions, regardless of the JP’s position. Upon a capture angle of 90°, where the left half of the JP serves as an insulator and the right half as a conductor, discontinuous transmembrane voltage distribution is noted at the lower cell vertex due to interaction with the upper JP vertex. Nevertheless, transmembrane voltage distribution remains continuous elsewhere, with positive and negative amplitude reaching approximately 0.8 V. Furthermore, the comparative transmembrane voltage distributions for bulk electroporation simulation and nanochannel-based electroporation simulation [46] have been plotted in Figure 3f. It is evident that under the same electroporation conditions, the conventional bulk electroporation method requires a higher pulse voltage to be applied, and the range between the electrode plates is larger, thus affecting the controllability of the electroporation operation. In contrast, nanochannel electroporation exhibits superior local effects and higher controllability over cell interactions.

### 3.3. Formation of Pore on Cell Membrane during Electroporation

**Figure 4:**
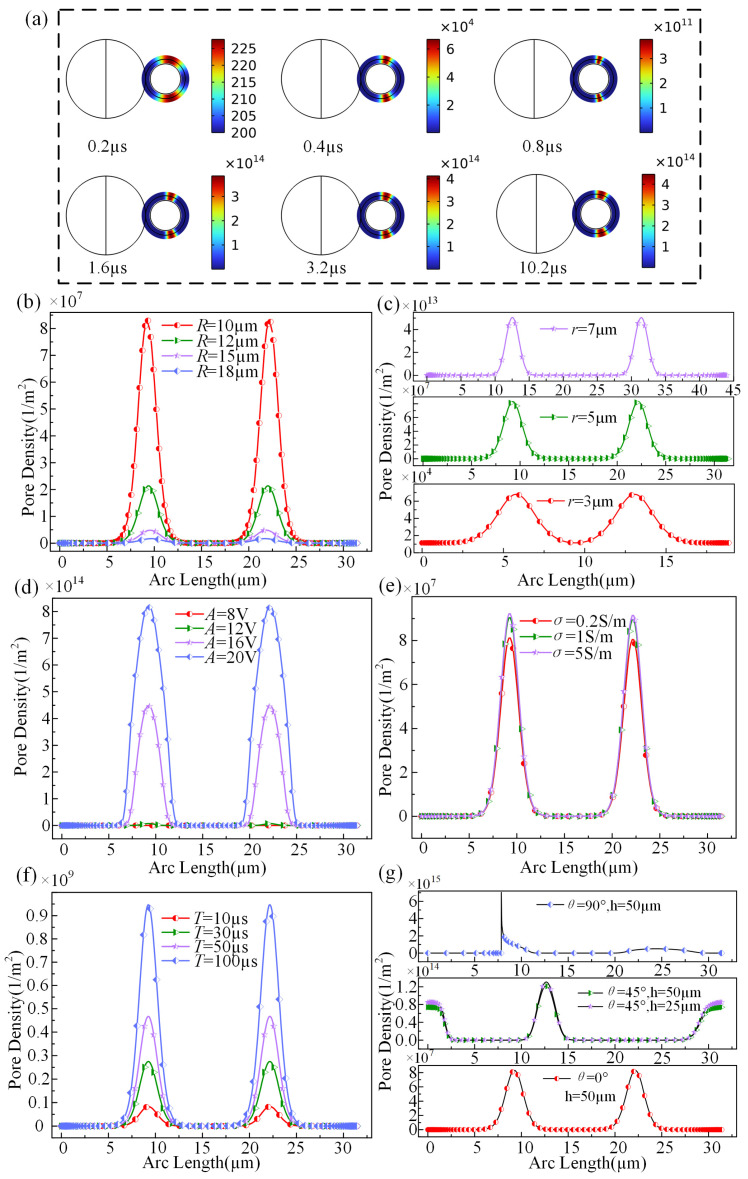
(**a**) The distribution of pore density at time instants of 0.2 µs, 0.4 µs, 0.8 µs, 1.6 µs, 3.2 µs, and 10.2 µs, respectively. (**b**) The pore density distribution curves for JP with radii of 10 µm, 12 µm, 15 µm, and 18 µm. (**c**) The pore density distribution curves for cell radii of 3 µm, 5 µm, and 7 µm, (**d**) the pore density distribution curves for pulse voltages of 8 V, 12 V, 16 V, and 20 V. (**e**) The pore density distribution curves for electrical conductivities of 0.2 S/m, 1 S/m, and 5 S/m. (**f**) The pore density distribution curves for pulse widths of 10 µs, 30 µs, 50 µs, and 100 µs. (**g**) The pore density distribution curves for Janus spheres capturing cell positions at 90° and at a distance of 25 µm from the electrode.

The impact of JPs on electroporation-induced pore density is investigated based on transmembrane voltage. This study categorizes the transmembrane voltage of cells and scrutinizes the factors influencing pore density across six dimensions: JP radius, cell radius, pulse voltage, conductivity, pulse width, and cell location, as illustrated in Figure 4. 

To better investigate the fluctuation in pore density distribution, this study analyzed the variation in pore density in cells at different time points within the same pulsing period. Figure 4a displays the time-resolved transmembrane voltage distribution of cells subjected to a pulse voltage of 16 V and pulse width of 10 µs. The application of an electrical pulse signal commences at 0.2 µs, leading to a slight increase in pore density on the cell surface due to the influence of JPs-induced potential. As the pulse progresses, the transmembrane voltage gradually rises, resulting in a substantial rise in pore density, reaching around 10^4^ at 0.4 µs. By 0.8 µs, the transmembrane voltage continues to escalate, leading to a notable electroporation effect and a corresponding pore density increase to around 10^11^. Subsequent electrical pulses further enhance the pore density, reaching levels around 10^14^ at 1.6 µs and 3.2 µs. Although the electroporation effect persists in subsequent pulse periods, the growth rate of pore density stabilizes around 10^14^. Notably, with JPs and cells located at the center of the electrode plate, symmetry is observed in the perforation density on the upper and lower cell surfaces. The variation in pore density is consistent with the transmembrane voltage distribution, where the maximum pore density aligns with the peak transmembrane voltage, confirming adherence to electroperforation principles in simulation outcomes. Figure 4b illustrates the pore density distribution curve corresponding to variations in JP size. The pore density exhibits a sharp decrease with the increasing radius of the JP. Subsequently, in Figure 4c, the pore density distribution curve showcases a notable increase as the cell radius expands, with the magnitude rising from 10^7^ initially to 10^14^. Different maximum pore density positions indicate the influence of JPs on the spatial distribution of cell pore density. The observed trends in pore density variation in Figure 4b,c align with the findings depicted in Figure 3b.

Figure 4d depicts the distribution curve of pore density as the pulse voltage changes. With the increase in pulse voltage, the pore density also increases. When the voltage difference is large, the order of magnitude of pore density varies significantly, consistent with the previously described distribution pattern of electroporation. Turning to Figure 4e, the pore density distribution curve under varied electrical conductivity levels indicates a consistent increase in pore density with rising electrical conductivity, albeit with minor fluctuations. Figure 4f shows the distribution curves of pore density under different pulse widths. With the increase in pulse width, the pore density also increases, reaching a magnitude of 10^9^, which is consistent with the research results of Mi Yan. This result reflects that the variation in pulse width does not affect the amplitude of transmembrane voltage but does affect pore density. Turning to observations in Figure 4g, it shows the distribution curves of electrically induced pore density in cells at different positions. When the capture angle is 0°, the pore density is minimal, with a magnitude of only 10^7^. At a capture angle of 45°, even with the change in the position of Janus spheres, the pore density distribution curves are similar and vary slightly between the two cases. At 90°, the pore density reaches its maximum magnitude of 10^15^ and is influenced by the vertices of the JP, exhibiting a discontinuous distribution at the bottom vertices of the cell. In all of these cases, the maximum pore density corresponds to the maximum values of transmembrane voltage and electric field strength, denoted as point A in Figure 1.

### 3.4. Electroporation of Chained Cells

**Figure 5:**
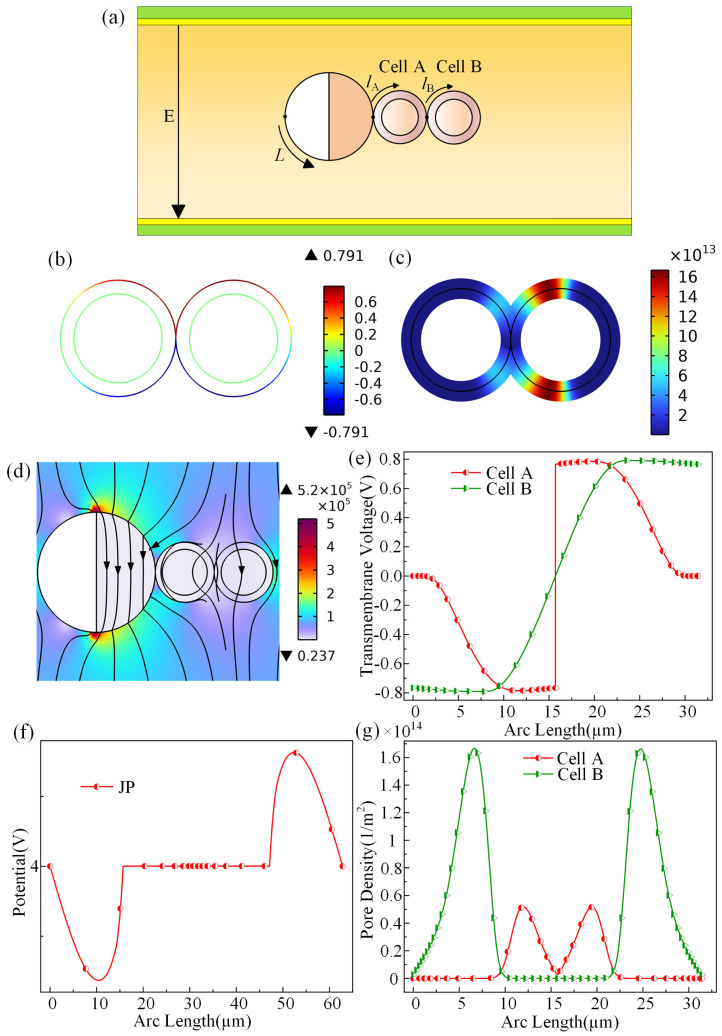
(**a**) Schematic diagram of electroporation when the JP captures two adherent cells. (**b**) Voltage distribution when the transmembrane voltage of cells A and B is at its maximum value. (**c**) Pore density distribution when the perforation density of cells A and B is at its maximum value. (**d**) electric field distribution when the JP captures two adherent cells by electroporation. (**e**) Curve of the transmembrane voltage of cells A and B. (**f**) Curve of the induced potential distribution of the JP. (**g**) Curve of the perforation density distribution of cells A and B.

This study examines the impact of electroporation on the cellular inner and outer membranes, encompassing factors such as the potential induced by JPs, transmembrane voltage, pore density, and perforation regions. Furthermore, during practical electroporation procedures, multiple cell chains always appear. To address this, an additional cell identical to the original model is introduced on the right side, simulating the adhesion of multiple cells under realistic conditions. The ensuing analysis delves into the electroporation effects of this setup, as depicted in Figure 5.

Figure 5a presents a simulation of a multicellular scenario, with cells denoted as A and B. Figure 5b illustrates the transmembrane voltage distribution of cell A and cell B. The figure reveals that the area with a larger distribution of transmembrane voltage values on cell B in the figure is slightly larger than that of cell A. The maximum transmembrane voltage occurs at the cell adhesion points, and the transmembrane voltage distribution of the two cells is approximately symmetric. In Figure 5c, a color-coded map displays the pore density distribution of cells A and B. The visual representation highlights significantly higher pore density in cell B, especially concentrated in the upper left and lower left areas. Analysis of the electric field diagram in Figure 5d demonstrates the impact of the JP on altering the electric field distribution near cell A, with minimal effect on cell B due to its middle separation. This change aligns with the observed transmembrane voltage and pore density distributions, explaining the higher pore density in cell B. Furthermore, Figure 5e depicts the symmetric distribution of transmembrane voltage across the cell surface, with a maximum value of 0.8 V. The induced electric potential distribution of Janus particles is shown in Figure 5f, and its distribution pattern is consistent with the rule described earlier in the text. Finally, Figure 5g showcases the pore density distribution curve on the cell surfaces of A and B. When correlated with Figure 5c, the data confirm the notably higher pore density in cell B, symmetric distribution, and the point of maximum pore density coincides with the peak transmembrane voltage and electric field intensity at point A.

In conclusion, optimizing pulse voltage in practical applications enhances cell transfection and DNA delivery, crucial for effective clinical treatments like cancer drug administration. Adjusting pulse duration stabilizes pores and regulates cell damage, ensuring therapeutic efficacy while sparing surrounding tissues. The role of media electrical conductivity in electroporation is pivotal for experimental condition selection, with its optimization in clinical settings boosting therapy effectiveness and control. Furthermore, the positioning of the JP and the radius of microelectrodes are critical for maximizing electroporation efficiency and contact with target cells or tissues, facilitating precisely targeted therapies and efficacy assessments in both laboratory and clinical environments. However, the movement control of JP microelectrodes, particularly within complex cellular environments, still presents unique challenges.

## 4. Conclusions

In this work, we have developed an in situ electroporation technique that capitalizes on the localized enhanced electric field generated around Janus particle (JP) microelectrodes within an ITO-sandwiched microfluidic chip. Through the development of a comprehensive simulation model, we have gained valuable insights into the dynamics of localized electroporation for both single cells and chained cells, highlighting the critical role of the electric field induced by Janus microelectrodes in cell membrane permeabilization. The systematic investigation of various parameters, including pulse voltage, duration, medium conductivity, JP position, and the radius of JP microelectrodes, has provided a deeper understanding of the factors influencing the electroporation effect. The results show that a wireless Janus particle (JP) microelectrode is strategically excited by an external electric signal applied to the indium tin oxide (ITO) electrodes. This stimulation results in the generation of an electric potential that significantly amplifies the electric intensity at the poles of the JP microelectrode, which leads to a distinct alteration in the electric field distribution surrounding the cells. Such precise manipulation of the electric field enables strategic control over the transmembrane voltage and nanopore formation, thereby facilitating spatially precise control over the intracellular delivery area. Furthermore, the differences in electroporation distribution among chain cells, as revealed by our simulations, provide insightful directives for the electroporation of tissues or cell populations. It is therefore believed that the JP electrode-based electroporation approach with the merits of versatile and precisely controllable modality can provide a promising tool for further contributing to the broader goal of cellular manipulation and personalized medicine.

## Data Availability

The original contributions presented in the study are included in the article, further inquiries can be directed to the corresponding author/s.
